# Leaf structure, physiology and transcriptome response of ancient *Populus szechuanica* cuttings in southwest China under different drought stresses

**DOI:** 10.1515/biol-2025-1310

**Published:** 2026-05-04

**Authors:** Zekun Zhang, Yanyuan Lu

**Affiliations:** Key Laboratory of Forest Resources Conservation and Utilization in the Southwest Mountains of China, Ministry of Education, Southwest Forestry University, Kunming, 650224, China; Key Laboratory for Conservation and Utilization of In-forest Resource of Yunnan, Southwest Forestry University, Kunming, 650224, China; Key Laboratory of Forest Disaster Warning and Control in Yunnan Province, Southwest Forestry University, Kunming, 650224, China

**Keywords:** ancient *P. szechuanica*, drought stress, transcriptome analysis, anatomical structure, physiology and biochemistry

## Abstract

Against the background of global climate change, increasingly severe drought stress exerts a significant impact on plant growth and yield. This study aimed to clarify the leaf anatomical structure, physiology and biochemistry and transcriptome-level metabolic adaptation mechanisms of ancient *P*. *szechuanica* to environmental stress. We selected cuttings of ancient *P. szechuanica* with a diameter of breast-height (DBH) ≥ 1 m and a tree age of 300–500 years as experimental materials. Natural drought stress was applied to investigate the responses of leaf anatomical structure, physiological and biochemical traits, and transcriptome-level metabolic processes of ancient *P. szechuanica* under drought stress. The results showed the following changes in leaf anatomical structure under drought stress (compared with the control group, the same below): leaf thickness, pith length and palisade tissue thickness decreased by 49.60 %, 20.1 % and 28.68 %, respectively. The thickness of upper and lower epidermis and spongy tissue first increased and then decreased, with final reductions of 53.13 %, 54.26 % and 50.30 %, respectively. Stomatal length and width also decreased, by 15.67 % and 24.26 % respectively. For physiological and biochemical traits, with the prolongation of drought stress, the soluble sugar content decreased significantly by 7.49 %, while the soluble protein content increased significantly by 44 %.At the transcriptome level, significant differentially expressed genes (DEGs) were screened at different drought stages: 3,353 upregulated and 3,161 downregulated DEGs on the day 4 of drought, 5,208 up-regulated and 9,560 down-regulated DEGs on the day 8, and 15,659 up-regulated and 14,870 down-regulated DEGs on the day 12. These DEGs mediated the drought stress response of *P. szechuanica* via positive up- or down-regulation.

## 1 Introduction

Against the backdrop of global climate and environmental changes, the seasonal and regional distribution of precipitation has become increasingly uneven, leading to more frequent droughts with broader impacts and greater severity. Water plays a crucial role in plant growth and development. Prolonged drought not only alters the external morphology of plants but also damages their physiological structures and biochemical metabolism. Severe drought stress can inflict irreversible damage on plants, ultimately leading to organ failure and even death [1]. Poplar trees are characterized by rapid growth, diverse genotypes, wide distribution, and suitability as material for molecular analysis [2]. The *P. szechuanica* belongs to the *Populus* genus within the Salicaceae family. It primarily grows in shaded, moist environments under forests or along forest edges at elevations between 1,100 and 4,600 m. Its distribution spans regions including Yunnan, Gansu, Sichuan, and Shaanxi. Its growth type is classified as temperate-humid, making it an excellent drought-resistant species and one of the most widely utilized tree species today. Due to unique geological and historical conditions, the southwestern region preserves abundant ancient poplar resources. These ancient trees have survived through the long-term combined effects of natural environmental factors and human activities, adapting to their local habitats. They harbor valuable genes related to longevity and stress resistance, and thus exhibit strong tolerance to adverse environmental conditions. However, due to the remote locations and harsh habitats of ancient poplars, comprehensive sampling has been challenging, leading to limited research on these ancient trees. Current studies primarily focus on resource collection and conservation, phylogenetic evolution, hybrid breeding, and anti-aging experiments, with relatively few investigations into their stress adaptation mechanisms. Therefore, in this study, cuttings from ancient *P. szechuanica* were used as experimental materials. By subjecting them to varying degrees of drought stress, we investigate their leaf anatomical structure, physiology and biochemistry, and transcriptional response mechanisms under different drought intensities. This research aims to provide a scientific basis for the utilization and development of ancient poplar resources in Southwest China, as well as for breeding drought-tolerant tree species in arid regions.

## 2 Materials and methods

### 2.1 Test site overview

The experimental site was located in the greenhouse of Southwest Forestry University at 25.05°N latitude and 102.75°E longitude, with an elevation of 1,980 m. It features a subtropical plateau monsoon climate. The annual average temperature was 16 °C, with a frost-free period exceeding 240 days and an annual precipitation of 1,035 mm.

### 2.2 Test materials

Within the primary distribution areas of poplar trees in Southwest China, representative *P. szechuanica* (breast height diameter >1 m, tree age 300–500 years) were sampled. Healthy one-year-old branches were collected and transplanted to the greenhouse of Southwest Forestry University for cutting propagation. After one year of growth, samples were collected for experimental analysis.

### 2.3 Drought management

Once the cuttings grew robust and stable, natural drought treatment was applied: thoroughly water the plants once before drought onset, then watering was completely withheld thereafter. Day 0 serves as the control. Sampling occurred every 4 days after treatment initiation until complete wilting of plants (in the day 12), with the entire drought process lasting 12 days. Collected samples (5 leaves per group) were partially used for leaf anatomical structure analysis, while the remainder were rapidly frozen in liquid nitrogen and stored at −80 °C for physiological, biochemical, and transcriptomic measurements. Each treatment comprised three replicates, with five plants per replicate, totaling 15 plants.

### 2.4 Experimental methods

#### 2.4.1 Paraffin section preparation and observation

Samples were removed from the FAA fixative, dehydrated with a gradient of alcohols, embed in paraffin, sectioned using a microtome, stained with hematoxylin and eosin stain, and mounted with neutral binder.

Observed the prepared sections under a Motic optical microscope. Selected paraffin sections with clear and intact structures for imaging using Motic Image Advanced 3.2 measurement software. Measured and recorded leaf morphological and anatomical parameters including stratum corneum thickness, upper epidermis thickness, lower epidermis thickness, leaf thickness, palisade tissue thickness, and leaf tissue structural compactness. Repeated measurements 10 times per field of view.

#### 2.4.2 Observation of stomata

Temporary slides were prepared via the nail polish imprint method and photographed, with 10 fields of view processed per slide. Subsequently, the length and width of the stomata were measured using ImageJ software.

#### 2.4.3 Measurement of physiological and biochemical indicators

Soil moisture content was measured using the TDR200 soil moisture meter (Spectrum, USA). Leaf relative water content was determined according to Hou’s method [3]. Soluble protein content was determined via the Coomassie Brilliant Blue G-250 method. Soluble sugars were measured by the anthrone colorimetric method.

#### 2.4.4 Transcriptome sequencing

The collected leaf samples were pooled, rapidly frozen in liquid nitrogen, and sent to Nomic Metabolomics for sequencing analysis. The paired-end library data, which had a read depth of 0.43 GB per sample and an alignment rate of 89.03 % against the v3.0 reference genome, was suitable for subsequent analysis.

### 2.5 Data analysis

Experimental data were processed using Excel 2016, and the processed data were statistically analyzed using SPSS 20. Transcriptomes were visualized using ggplots2 software to generate volcano plots of differentially expressed genes, with genes meeting the criteria of |log2fold change| >1 and P-value <0.05 designated as differentially expressed genes. GO enrichment analysis was performed using topGO software. Calculate the gene list and gene count for each GO term annotated to the differentially expressed genes. *P-values* were calculated using the hypergeometric distribution method (significance threshold: *P*-value <0.05). Identify genes significantly enriched relative to the whole-genome background. Conduct GO and KEGG enrichment analyses to determine the primary biological functions of differentially expressed genes (all data included multiple-testing correction).

## 3 Results and analysis

### 3.1 Effects of drought stress duration on soil moisture content and leaf relative water content

**Figure 1:**
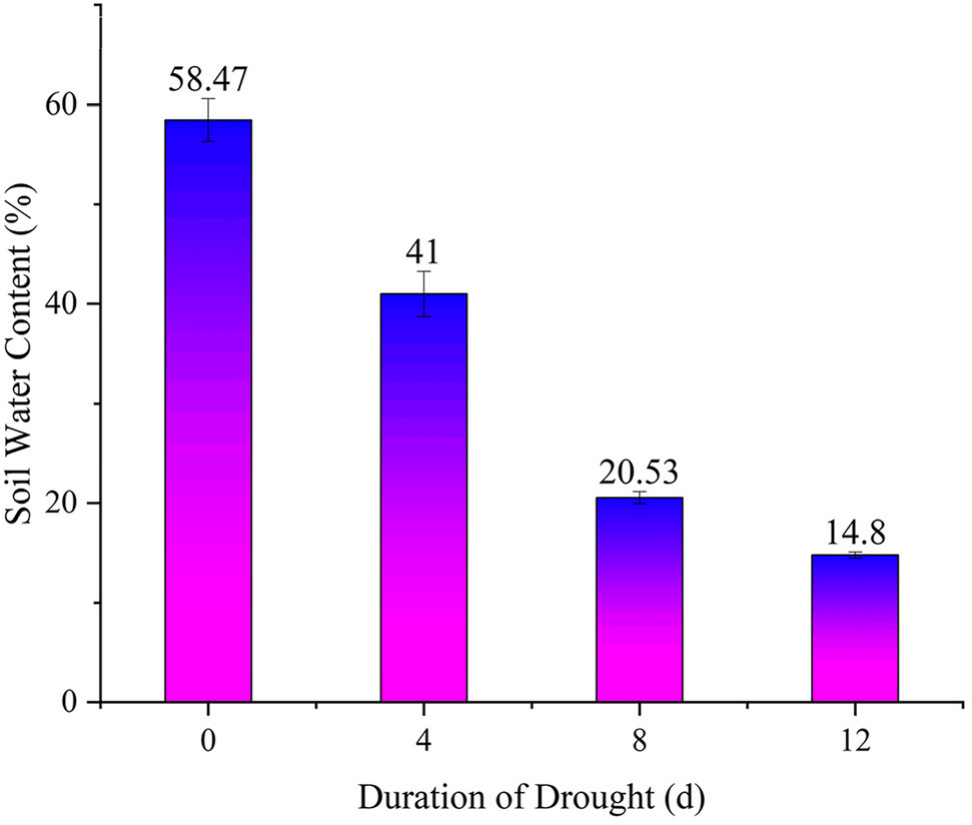
Changes in soil water content of *P. szechuanica* cuttings under drought stress day 0, day 4, day 8 and day 12. Different letters indicate significant differences among treatments for *P. szechuanica* cuttings (P < 0.05). The same applies below.

As shown in Figure 1, compared to day 0 (the same bellows), the soil moisture content of *P. szechuanica* cuttings exhibited a significant downward trend as drought stress intensified. By day 12, soil moisture had dropped markedly to approximately 15 %, representing a 75 % reduction compared with the control group. This indicated that soil moisture had reached the minimum threshold at this stage.

**Figure 2:**
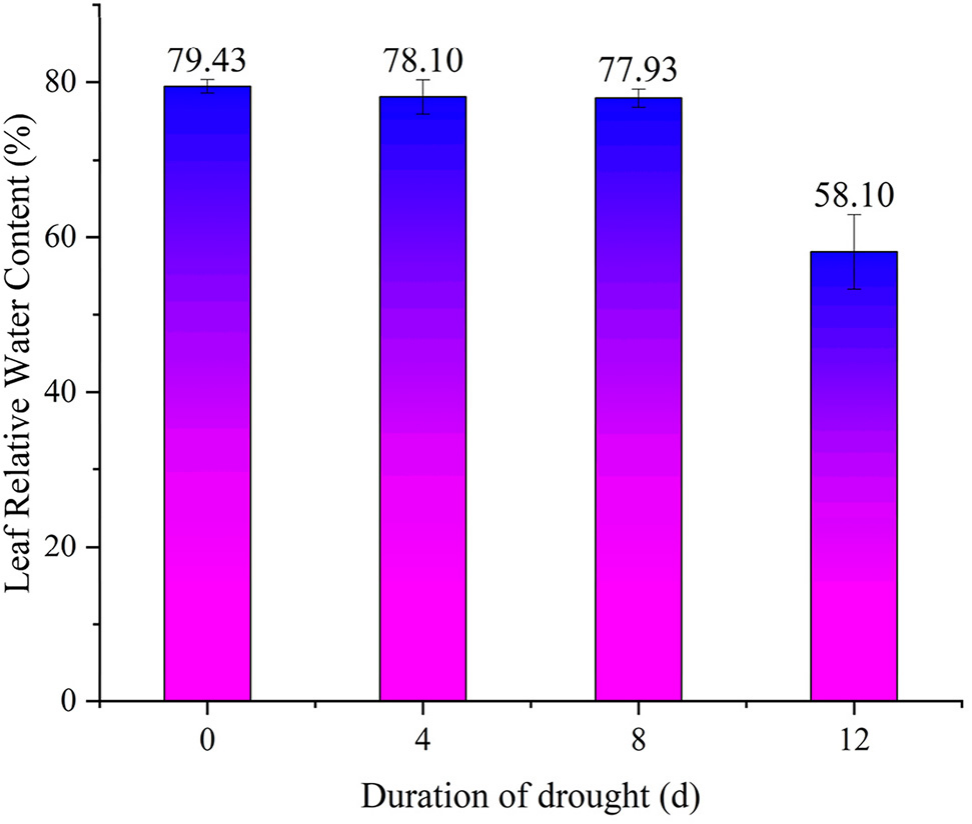
Changes in relative leaf water content of *P. szechuanica* cuttings under drought stress day 0, day 4, day 8 and day 12.

As shown in Figure 2, the relative leaf water content of *P. szechuanica* cuttings declined gradually overall during the natural drought stress period. However, the change in relative leaf water content was not significant during the early to mid-stage of drought (day 0 to day 8). On the day 12 of drought, the relative leaf water content dropped to 58.10 %. The relative leaf water content of the control group was 79.43 %. This meant a reduction of 26.9 % in the drought group. This decrease was significantly different from that of the control group (P < 0.05). These results indicate that *P. szechuanica* leaves can maintain a relatively high water content under a certain degree of drought. Once the drought stress exceeds a certain threshold, severe water loss occurs in the leaves. This severe leaf water loss leads to the impairment of leaf function (included multiple-testing correction, the same bellows).

### 3.2 Effects of drought stress duration on leaf anatomical structure of cuttings from ancient *P. szechuanica*

**Figure 3:**
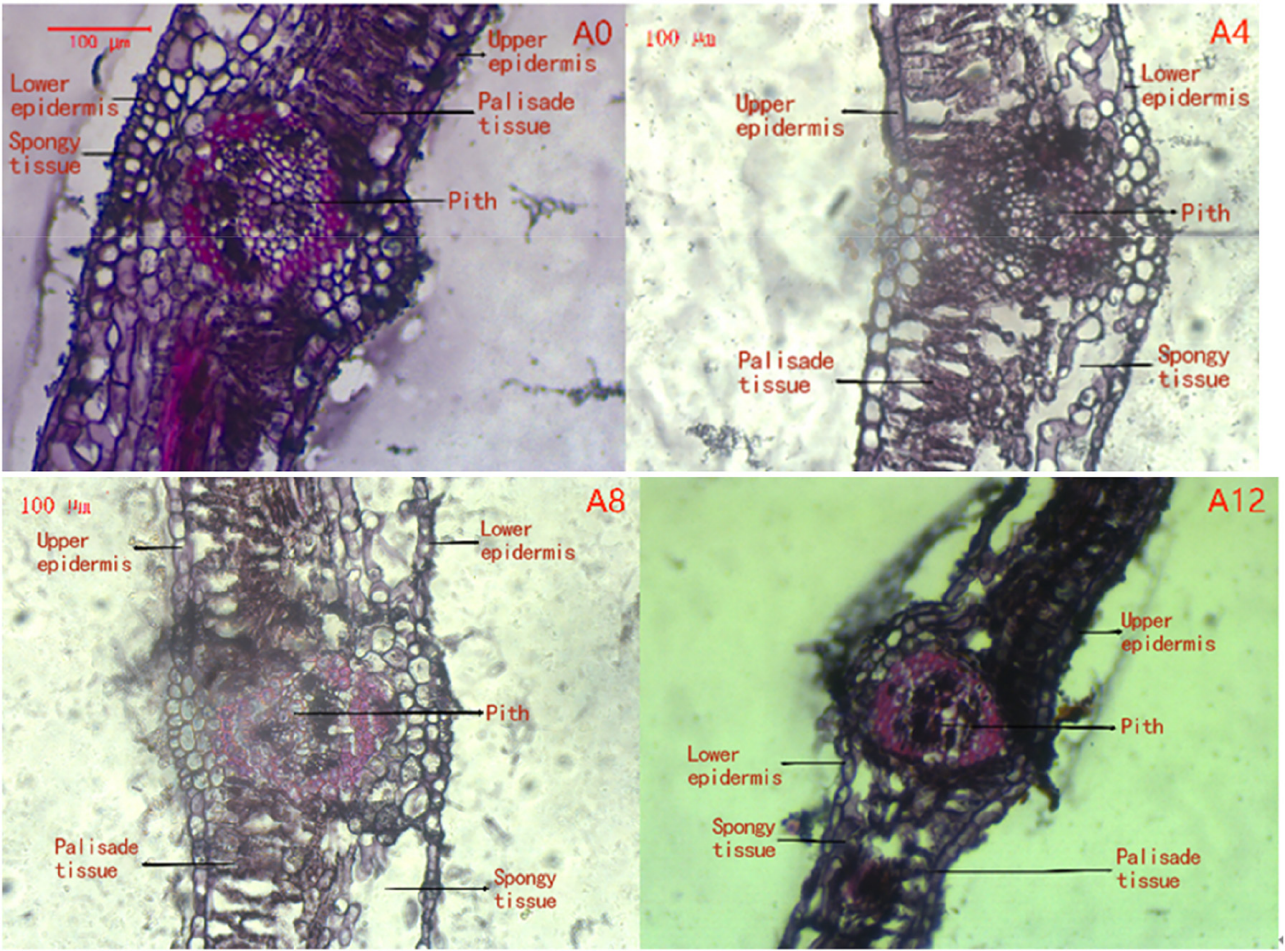
Changes in leaf anatomy of *P. szechuanica* under drought stress day 0, day 4, day 8 and day 12.

Under drought stress, the most obvious response of plants is leaf yellowing, wilting, drooping and curling. The anatomical structure of poplar leaves changes significantly under soil drought stress, as shown in Figure 3. At the initial stage of drought, leaf cells are plump and clearly defined. Palisade and spongy tissues are completely intact (Figure 3, A0). On the day 4 and day 8 of drought stress, partial leaf cell death occurs. The thickness of palisade and spongy tissues decreases. The cells become more closely packed (Figure 3, A4, A8). On the day 12 of drought stress, most leaf cells die. The upper and lower epidermal layers become structurally incomplete. The thickness of palisade and spongy tissues drops sharply (Figure 3, A12). These observations indicate that drought severity is positively correlated with the degree of change in poplar leaf anatomical structure. It also means that the physiological functions of poplar leaves gradually weaken with the intensification of drought stress.

Drought is one of the major abiotic stresses that impairs plant growth and development and severely restricts crop yield and geographical distribution. It severely limits crop yields and their geographical distribution. As shown in Table 1, multiple leaf anatomical indices decreased significantly. These indices are leaf thickness, pith length, upper and lower epidermis thickness, palisade tissue thickness and spongy tissue thickness. Among them, the reduction in pith length was notably obvious. Compared with the control group, the leaf thickness of *P. szechuanica* decreased by 49.60 % on the day 12 of drought. This difference was statistically significant. The thickness of upper epidermis, lower epidermis and palisade tissue first increased and then decreased. But the increase in lower epidermis thickness was not pronounced. Spongy tissue thickness showed a continuous decline during drought stress. The ratio of palisade tissue/spongy tissue also first increased and then decreased. It reached the peak on the day 8 day of drought, then declined afterwards. Under drought stress, this ratio in *P. szechuanica* remained above 1. This indicates that the species responds to drought impacts by regulating the thickness of its water-storage tissues. These water-storage tissues are the palisade layers and spongy layers.

**Table 1:** Changes in leaf morphology of *P. szechuanica* cuttings under drought day 0, day 4, day 8 and day 12.

	Day 0	Day 4	Day 8	Day 12
Leaf thickness (μm)	274.37 ± 8.19a	251.87 ± 14.77b	228.50 ± 1.72c	138.27 ± 7.72d
Pith length (μm)	152.40 ± 4.20a	126.77 ± 11.78b	125.73 ± 4.92b	121.37 ± 3.70b
Upper epidermis thickness (μm)	17.07 ± 1.20b	21.63 ± 0.88a	19.07 ± 1.13ab	8.00 ± 1.77c
Lower epidermis thickness (μm)	15.37 ± 1.02a	16.72 ± 0.54a	16.50 ± 0.70a	7.03 ± 1.33b
Palisade tissue thickness (μm)	96.33 ± 3.39a	129.50 ± 4.63b	120.83 ± 2.49c	68.70 ± 1.71d
Spongy tissue thickness (μm)	92.70 ± 1.85a	76.50 ± 3.56b	68.47 ± 1.35c	46.07 ± 4.91d
The palisade/spongy tissue ratio	1.04	1.69	1.76	1.49

Different letters in the table indicate significant differences between treatments (P < 0.05).

### 3.3 Effects of drought stress duration on leaf stomatal of cuttings from ancient *P. szechuanica*

Drought stress has significant effects on leaf stomata. Table 2 shows that stomatal length varied with increasing drought stress intensity. It first decreases and then increases during the drought period. Compared to the day 0 stomatal length decreased significantly on the day 4. It increased slightly on the 8th day and decreased slightly again on the day 12. There were no significant differences in stomatal length among the day 4, day 8 and day 12 treatments. As for stomatal width, it decreased gradually as drought stress intensified. Stomatal width on the day 8 and day 12 differed significantly from that on the day 0. But there was no significant difference in stomatal width between the day 8 and day 12. These results suggested that ancient *P. szechuanica* modulates stomatal traits to conserve water, thereby adapting to drought stress.

**Table 2:** Changes in leaf stomatal of *P. szechuanica* cuttings under drought day 0, day 4, day 8 and day 12.

Treatment	Stomatal parameters	
	Stomatal length (μm)	Stomatal width (μm)
Day 0	28.47 ± 4.84a	22.01 ± 2.00a
Day 4	24.49 ± 3.09b	20.86 ± 2.21a
Day 8	26.33 ± 2.34ab	18.40 ± 1.81b
Day 12	24.01 ± 1.73b	16.67 ± 2.66b

Different letters in the table indicate significant differences between treatments (P < 0.05).

### 3.4 Effects of drought stress duration on soluble sugar and soluble protein content in leaves of cuttings from ancient *P. szechuanica*

**Figure 4:**
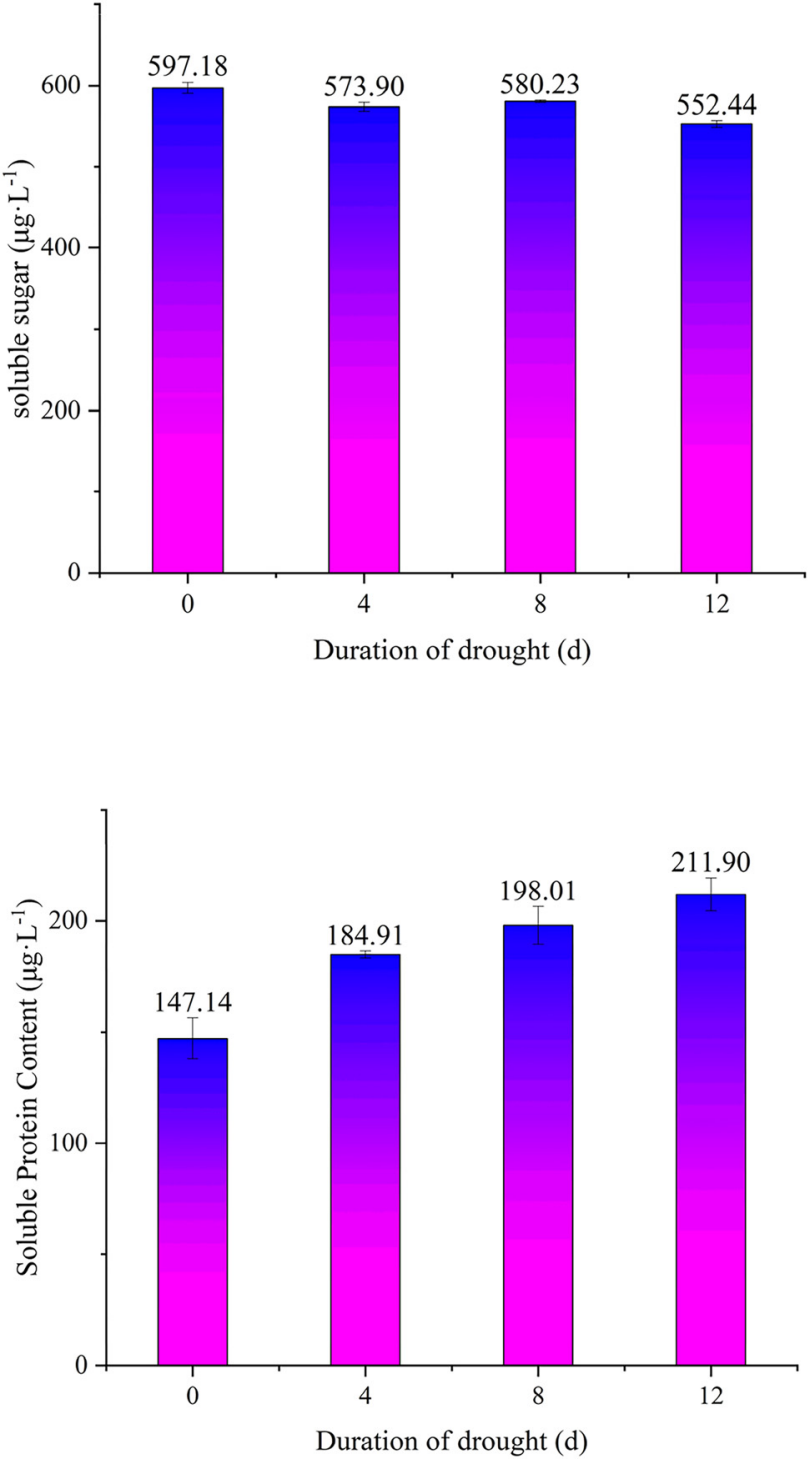
Changes of leaf soluble sugar and soluble protein contents under different drought stress day 0, day 4, day 8 and day 12.

As shown in Figure 4, soluble sugar content in *P. szechuanica* leaves varied with extended drought duration. It showed a trend of first decreasing and then increasing. On the day 4 of natural drought stress, the leaf soluble sugar content decreased significantly compared to the control. The reduction was 4.5 %, and this difference was statistically significant (P < 0.05). On the day 8 of drought stress, the soluble sugar content first rose slightly. Then it declined significantly afterwards. On the day 12 of drought stress, the soluble sugar content dropped to only 552.44 μg L^−1^. These results indicate that soluble sugars can effectively maintain cellular water content only within a certain threshold.

When drought stress exceeds a certain intensity, the osmotic regulatory function of soluble sugars is inhibited. Under drought stress, the soluble protein content in the leaves of *P. szechuanica* cuttings showed a continuous increasing trend. Compared with the control group, the leaf soluble protein content increased by 25.7 % on the 4th day of drought. This difference was statistically significant (P < 0.05). On the 12th day of drought, the soluble protein content in the leaves rose to 211.9 μg L^−1^. The soluble protein content of the control group was 147.14 μg L^−1^. This represented an increase of 44 %. These results indicate that soluble proteins in the leaves of *P. szechuanica* cuttings play a more significant role in the later stages of drought stress.

### 3.5 Effects of drought stress duration on transcriptome levels in leaves of cuttings from ancient *P. szechuanica*

#### 3.5.1 Transcriptome data quality control analysis

High-throughput sequencing was performed on all samples. The related sequencing data are shown in Table 3. The total base counts at day 0, day 4, day 8 and day 12 were 6,492,340,533 bp, 6,564,634,803 bp, 6,285,022,667 bp and 6,285,843,503 bp, respectively. The total read counts at these four time points were 42,995,633 bp, 43,474,403 bp, 41,622,667 bp and 41,628,103 bp, respectively. The total number of bases with an identification accuracy over 99.9 % was 6,044,982,528 bp, 6,100,605,130 bp, 5,864,890,257 bp and 5,887,399,261 bp at day 0, day 4, day 8 and day 12, respectively. The proportion of ambiguous bases at the four time points was 0.001728 %, 0.001763 %, 0.001684 % and 0.001477 %, respectively. The percentage of bases with an identification accuracy above 99 % reached 97.63 %, 97.55 %, 97.72 % and 97.81 % at day 0, day 4, day 8 and day 12, respectively. The percentage of bases with an identification accuracy above 99.9 % was 93.10 %, 92.90 %, 93.32 % and 93.64 % at the corresponding time points, respectively. These results indicate that the cDNA library constructed and sequenced in this study is of high quality. This high-quality cDNA library is suitable for subsequent bioinformatics analysis.

**Table 3:** Transcriptome sequencing output quality statistics.

Sample	Reads no.	Bases (bp)	Q30 (bp)	*N* (%)	Phred >20 Q20 (%)	Phred >30 Q30 (%)
CY0d-1	43,081,176	6,505,257,576	6,045,182,092	0.001683	97.55	92.92
CY0d-2	42,821,984	6,466,119,584	6,048,139,755	0.001707	97.81	93.53
CY0d-3	43,083,738	6,505,644,438	6,041,625,736	0.001794	97.52	92.86
CY4d-1	44,462,712	6,713,869,512	6,285,382,822	0.001766	97.85	93.61
CY4d-2	45,807,064	6,916,866,664	6,427,503,359	0.001876	97.55	92.92
CY4d-3	40,153,432	6,063,168,232	5,588,929,208	0.001648	97.24	92.17
CY8d-1	40,910,698	6,177,515,398	5,782,840,783	0.001695	97.83	93.61
CY8d-2	40,442,954	6,106,886,054	5,703,508,981	0.001697	97.77	93.39
CY8d-3	43,514,348	6,570,666,548	6,108,321,007	0.001661	97.56	92.96
CY12d-1	43,411,726	6,555,170,626	6,142,134,063	0.001305	97.84	93.69
CY12d-2	38,157,858	5,761,836,558	5,373,627,633	0.001682	97.67	93.26
CY12d-3	43,314,724	6,540,523,324	6,146,436,088	0.001445	97.93	93.97

#### 3.5.2 Differential gene expression analysis in leaves of *P. szechuanica* cuttings under drought stress

**Figure 5:**
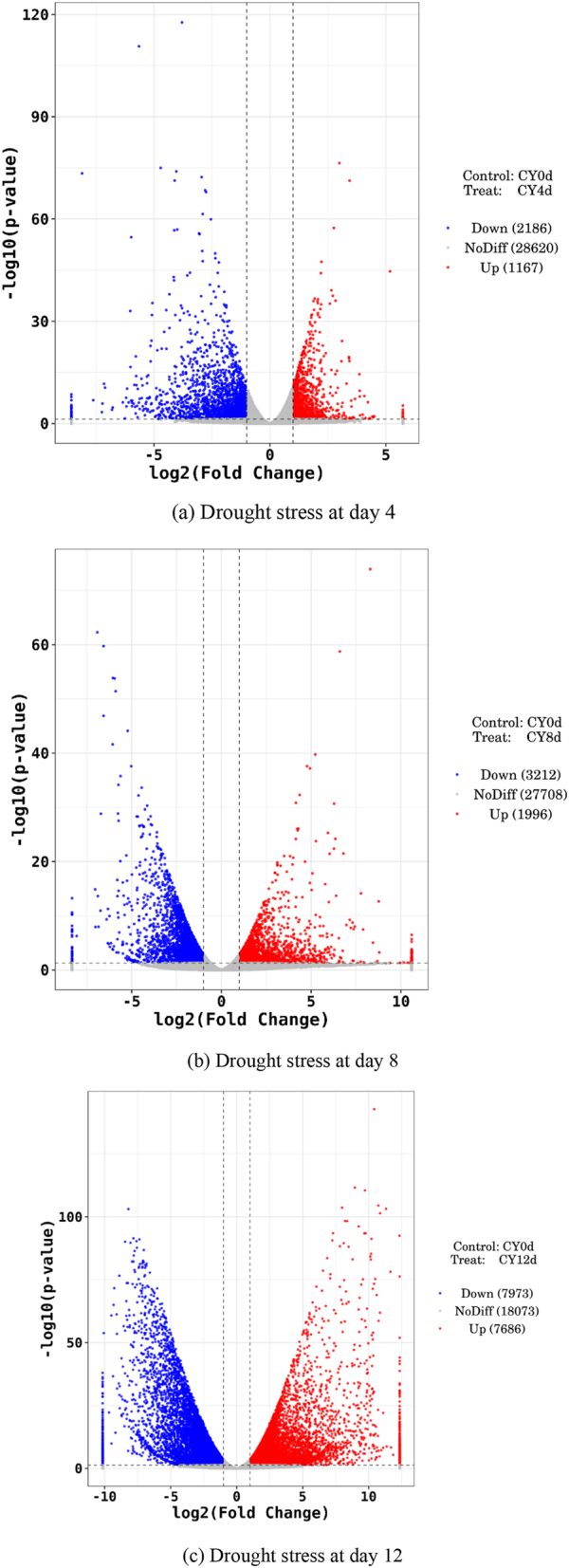
Transcriptome differential gene expression number of *P. szechuanica* leaves under drought stress day 4, day 8 and day 12.

Figure 5 and Table 4 results indicate that compared to the day-0 (0 d), drought stress at day 4 (0 d-vs-4 d) induced 3,353 differentially expressed genes (DEGs) in *P. szechuanica* leaves, comprising 1,167 upregulated genes and 2,186 downregulated genes. After day 8 of drought stress compared to day 0 (day 0-vs-day 8), a total of 5,208 genes exhibited altered expression, with 1,996 genes upregulated and 3,212 genes downregulated. Compared to day 0 (day 0 vs-day 12), drought stress at day 12 induced changes in 15,659 genes, with 7,686 genes upregulated and 7,973 genes downregulated. Thus, as drought stress duration increased, the number of DEGs in *P. szechuanica* leaves rapidly increased, with the number of downregulated DEGs was significantly higher than that of upregulated DEGs. Among these, the number of genes associated with drought resistance also increased significantly.

**Table 4:** Number of differentially expressed genes (DEGs) in *P. szechuanica* leaves under drought stress day 0, day 4, day 8 and day 12.

CK	Drought management	Upregulated genes	Downregulated genes	Total
Day 0	Day 4	1,167	2,186	3,353
Day 0	Day 8	1,996	3,212	5,208
Day 0	Day 12	7,686	7,973	15,659

#### 3.5.3 GO functional annotation and enrichment analysis of differentially expressed genes

**Figure 6:**
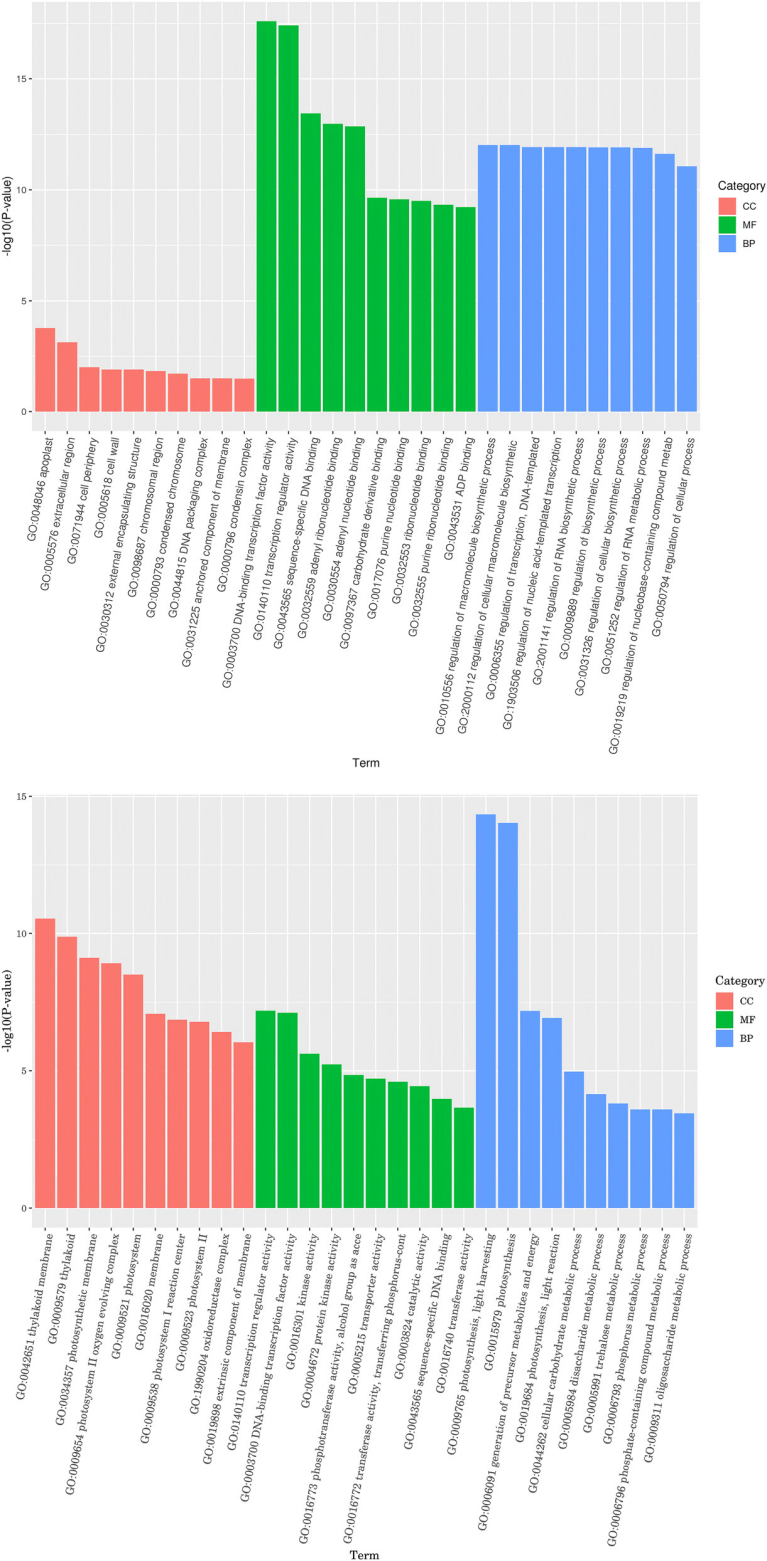

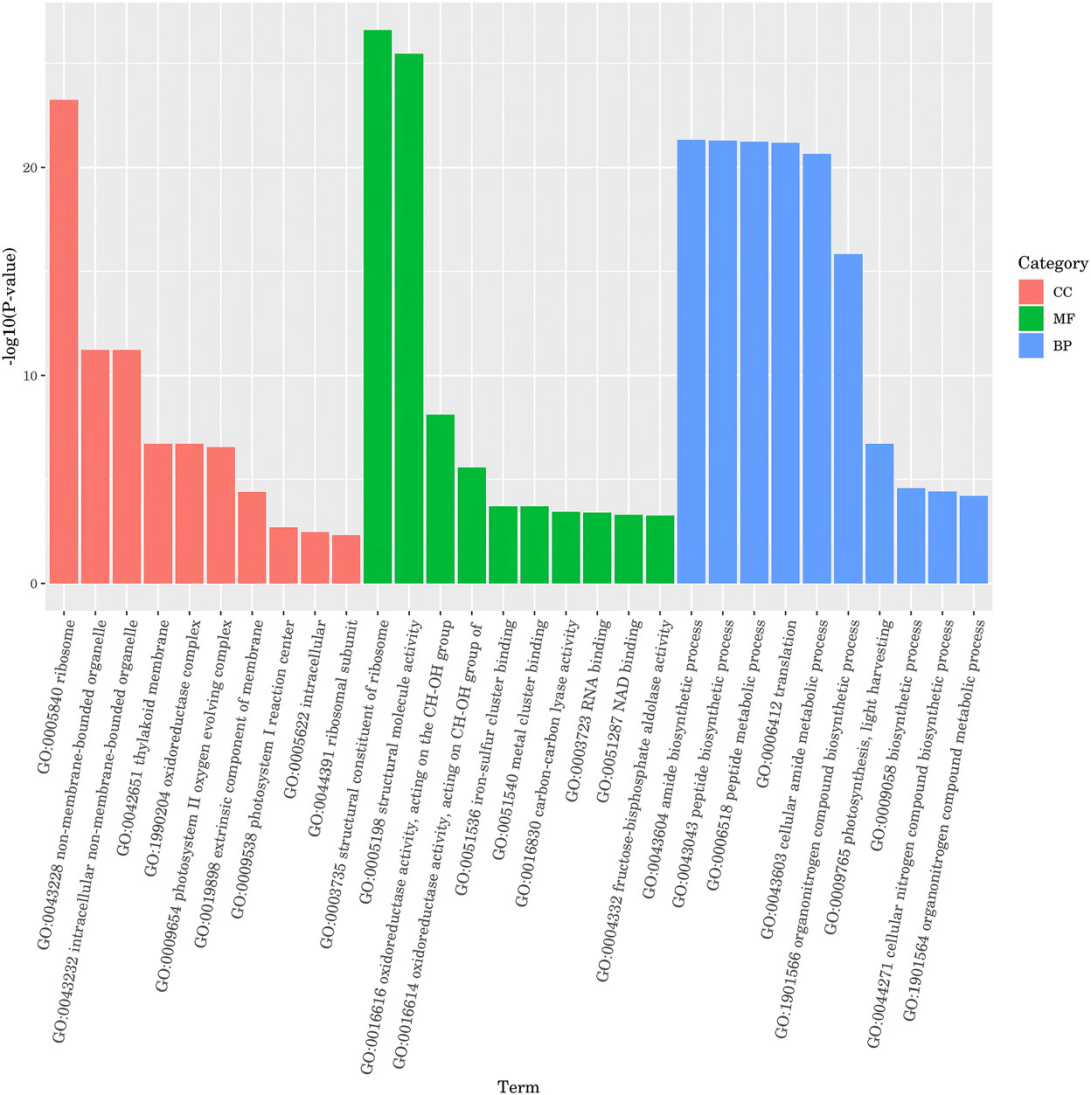
GO classification of differential genes in *P. szechuanica* cuttings.

GO functional annotation showed that DEGs were mainly enriched in three major categories: Molecular Function (MF), Biological Process (BP), and Cellular Component (CC) (Figure 6, Table 5). In the MF category, the top five most enriched metabolic pathways were identified. They were ADP binding, adenyl nucleotide binding, oxidoreductase activity, transcription regulator activity, and DNA-binding trandifferscription factor activity. In the BP category, the most enriched metabolic pathways were protein phosphorylation, peptide biosynthetic process, photosynthesis, translation, and disaccharide metabolic process. In the CC category, the most significant metabolic pathways were extracellular region, intrinsic component of membrane, integral component of membrane, ribosome, and thylakoid membrane. Under natural drought stress, three categories showed the greatest changes in gene numbers. These categories were oxidoreductase activity, adenylate binding, and protein phosphorylation. Furthermore, the number of DEGs in these categories increased with the prolongation of drought duration. It can thus be inferred that these physiological activities alter the leaf structure of plants. These activities also enhance the drought tolerance of plants.

**Table 5:** Classification of GO enrichment of differentially expressed genes in *P. szechuanica* cuttings.

Categories	GO annotation	d 0 vs d 4		d 0 vs d 4		d 0 vs d 12	
		U	D	U	D	U	D
MF	ADP binding	7	85	9	85	35	186
	Adenyl nucleotide binding	79	266	141	269	418	705
	Oxidoreductase activity	124	168	124	215	438	441
	Transcription regulator activity	41	92	71	74	152	139
	DNA-binding transcription factor	41	88	68	71	144	133
BP	Protein phosphorylation	55	184	142	197	346	466
	Peptide biosynthetic process	3	7	10	36	283	88
	Photosynthesis	2	1	1	65	9	82
	Translation	3	7	9	36	281	87
	Disaccharide metabolic process	3	5	13	5	21	10
CC	Extracellular region	3	12	8	17	17	18
	Intrinsic component of membrane	33	71	76	101	270	290
	Integral component of membrane	33	67	77	99	273	247
	Ribosome	2	4	6	23	246	49
	Thylakoid membrane	0	1	0	23	3	30

U, upregulated; D, downregulated.

#### 3.5.4 KEGG functional annotation and metabolic pathway enrichment analysis of differentially expressed genes

KEGG enrichment analysis revealed that DEGs in poplar leaves were mainly enriched in three major functional categories: metabolism, genetic information processing, and biological systems (Table 6), with the enriched pathways including environmental adaptation, signal transduction, carbohydrate metabolism, energy metabolism, metabolism of other amino acids, biosynthesis of other secondary metabolites, cofactor and vitamin metabolism, and terpenoid and polyketide metabolism. The number of DEGs in these pathways increased consistently with the prolongation of drought stress, and carbohydrate metabolism, signal transduction and energy metabolism were the three pathways enriched across the d 0 versus d 4, d 0 versus d 8, d 0 versus d 12 and d 0 versus d 8 versus d 12 comparisons. Distinct enrichment characteristics were observed under different drought durations: at the early drought stage (d 0 vs 4 d), carbohydrate metabolism (104 DEGs) and environmental adaptation (74 DEGs) showed the highest enrichment levels; at the mid-drought stage (0 d vs 8 d), energy metabolism (145 DEGs), signal transduction (141 DEGs) and carbohydrate metabolism (129 DEGs) were the top three enriched pathways; at the late drought stage (0 d vs 12 d), the number of DEGs increased significantly, with the most highly enriched pathways being carbohydrate metabolism (315 DEGs), signal transduction (285 DEGs), energy metabolism (258 DEGs) and biosynthesis of other secondary metabolites (233 DEGs). In summary, signal transduction, carbohydrate metabolism and energy metabolism were the core pathways mediating the drought stress response of poplar leaves, while the enrichment of specific pathways varied with drought stress intensity, with environmental adaptation being prominent in the early stage and biosynthesis of other secondary metabolites highly enriched in the late stage of drought.

**Table 6:** Enrichment analysis of KEGG signaling pathway of differentially expressed genes in *P. szechuanica* leaves.

KEGG signaling pathways	Number of differentially expressed genes		
	d 0 vs d 4	d 0 vs d 8	d 0 vs d 12
Environmental adaptation	74	60	163
Signal transduction	32	141	285
Carbohydrate metabolism	104	129	315
Energy metabolism	31	145	258
Metabolism of other amino acids	36	84	121
Biosynthesis of other secondary metabolites	39	65	233
Metabolism of cofactors and vitamins	23	72	104
Metabolism of terpenoids and polyketides	34	48	125

## 4 Discussion and conclusions

### 4.1 Discussion

Drought stress is the primary abiotic factor among the various environmental stresses affecting plants, severely limiting their growth, development, and distribution range. Under drought stress, plant roots produce stress signals and transport them to stem and leaf organs. These signals then trigger a series of structural and physiological biochemical changes to adapt to drought conditions. Therefore, observing anatomical structures provides a direct visual indication of the extent to which plants are affected under stress. Under water stress conditions, roots struggle to absorb water, leaf area decreases, and leaf water content declines. To minimize water loss, stomatal aperture inevitably reduces or even closes, thereby also affecting the degree of stomatal opening [4]. This indicates that stomatal width is significantly affected under drought stress in plants. The study found that under drought stress, both leaf thickness and pith length continuously decreased, which indicates plants can reduce transpiration by decreasing stomatal tension or closing stomata, thereby minimizing water loss and improving water use efficiency. This result is consistent with the findings of Farquhar et al. [5]. Palisade and spongy tissues thicken during the early stages of drought, enhancing leaf photosynthetic efficiency and water storage capacity while increasing water transport resistance and distance within the leaf. This partially mitigates water loss. However, both tissues significantly decline during the later stages of drought, likely due to severe water deficit within the plant. This impedes mesophyll cell division and growth, resulting in reduced cell numbers and volumes. These findings align with Shipley’s [6] research on *Populus simonii.* Bacelar et al. [7] also observed that under drought stress, plant leaf thickness decreased while the palisade and spongy tissues thickened.

Soluble sugars and soluble proteins are common osmoregulatory substances that stabilize cellular osmotic pressure, protect cell membrane structure, and enhance root water uptake capacity. In this study, soluble protein content gradually increased with prolonged stress duration. It is obvious that *P. szechuanica* downregulates genes in the protein degradation pathway to promote soluble protein accumulation. This mechanism facilitates expansion of leaf water-storage tissues, consistent with findings in *Lycium ruthenicum* [8], *Arachis hypogaea* [9], *Glycine max* [10], 11], and *Dendrobium officinale* [12] during drought stress. Another osmotic regulator, soluble sugar content, gradually decreased under drought stress. This finding differs from the conclusions of Lin and Chao [13], Wang et al. [14], Huang et al. [15], and Zhao et al. [16]. The reason lies in the upregulation of most genes in the soluble sugar degradation pathway inhibited *de novo* soluble sugar synthesis and accelerated its degradation.

Transcriptomics acts as a bridge linking genomic genetic information to proteomic functional information and serves as a key tool for understanding gene expression regulation in organisms [17]. This study performed transcriptome sequencing on leaves of *P. szechuanica* cuttings under different drought durations, identifying 3,353, 5,208, and 15,459 differentially expressed genes (DEGs) respectively – consistent with findings by Qian et al. [18], Wei et al. [19], Wang et al. [20], Wang et al. [21], Mulozi et al. [22], and Ren et al. [23], indicating that increasing drought intensity induces more DEGs to counteract adverse stress damage. GO analysis showed DEGs were mainly involved in DNA-binding transcription factor activity, transcription factor activity, photosynthesis, light energy absorption, ribosomes, and protein synthesis. KEGG analysis revealed significant enrichment of DEGs in signal transduction [24], [25], [26], carbohydrate metabolism [25], 26], MAPK signaling pathway [24], 27], 28], lipid metabolism [24], 28], amino acid metabolism [24], 28], 29], energy metabolism [24], 30], 31], ribosomes and protein synthesis [24], 31], and cell membranes and transport [26], 28] – pathways crucial for plant growth, development, and stress responses, consistent with Janiak et al. [32]. Deng et al. [33], Thayale Purayil et al. [34], and Bashir et al. [35] also reported that drought-induced DEGs are primarily enriched in pathways related to protein synthesis (e.g., endoplasmic reticulum protein processing), which aligns with the present study. Additionally, sequencing results showed DEGs in ancient *P. szechuanica* cuttings mainly participate in ribosome synthesis, consistent with Sharma et al. [36]. Previous studies have noted increased amino acid metabolism (e.g., alanine and glutamate metabolism) under drought, suggesting plants enhance relevant metabolic pathways through gene regulation to cope with stress.

### 4.2 Conclusions

Under drought stress, cuttings of ancient *P. szechuanica* reduce water loss through multiple morphological and physiological mechanisms Downregulation of genes in the protein degradation pathway leads to the accumulation of soluble proteins, an important osmotic regulator, thereby enhancing drought adaptation. Transcriptome analysis has revealed that more genes showed differential expression under more severe drought conditions. This suggested that *P. szechuanica* modulates its growth, stomatal movement, and leaf anatomical structure via DEG expression to resist dehydration and enhance drought tolerance. These mechanisms collectively functioned to resist plant dehydration and enhance drought tolerance.

## References

[1] Huang YY, Deng MH, Peng CX, Li J, Wang Y, Zhao Q (2020). Response of antioxidant enzyme system in lily petals to drought stress. Acta Hortic Sin.

[2] Sloan JL, Burney OT, Pinto JR, Garcia R, Miller S, Davis K (2020). Drought-conditioning modifies seedling anatomy and physiology of quaking Aspen (*Populus tremuloides* michx). Front Plant Sci.

[3] Hou FL (2018). Plant physiology experiments: a tutorial.

[4] Liu J, Zhang S, Chen L, Yang W, Zhao H, Zhu Y (2021). Effects of drought stress on leaf anatomical structure of ancient trees. Plant Ecol Sin.

[5] Farquhar GD, Sharkey TD (1982). Stomatal conductance and photosynthesis. Annu Rev Plant Physiol.

[6] Shipley B, Lechowicz MJ, Wright I, Reich PB (2006). Fundamental trade-offs generating the worldwide leaf economics spectrum. Ecology.

[7] Bacelar EA, Correia CM, Moutinho-Pereira JM, Gonçalves BC, Lopes JI, Torres-Pereira JM (2004). Sclerophylly and leaf anatomical traits of five field-grown olive cultivars growing under drought conditions. Tree Physiol.

[8] Zhang JJ, Tian Q, Guo YY, Zhang HY, Bao XG, Kong DS (2023). Physiological mechanisms of *Lycium ruthenicum* root system response to drought stress. J Gansu Agric Univ.

[9] Liu Y, Shen Y, Liang M, Zhang X, Xu J, Shen Y (2022). Identification of peanut AhMYB44 transcription factors and their multiple roles in drought stress responses. Plants.

[10] Iqbal N, Hussain S, Raza MA, Yang CQ, Safdar ME, Brestic M (2019). Drought tolerance of soybean (*Glycine max* L. Merr.) by improved photosynthetic characteristics and an efficient antioxidant enzyme activities under a split-root system. Front Physiol.

[11] Zhou Y, Li H, Chen H, Yang X, Yu T, Wang Y (2022). Proteomic investigation of molecular mechanisms in response to PEG-induced drought stress in soybean roots. Plants.

[12] Yang QY, Luo YZ, Yang Y, Ruan BL, Huang JM (2023). Effects of drought stress on physiological characteristics and active components in different parts of Dendrobium officinale. Jiangsu Agric Sci.

[13] Lin PH, Chao YY (2021). Different drought-tolerant mechanisms in quinoa (*Chenopodium quinoa* willd.) and djulis (*Chenopodium formosanum* koidz.) based on physiological analysis. Plants.

[14] Wang MZ, Li P, Li C, Pan Y, Jiang X, Zhu D (2014). SiLEA14, a novel atypical LEA protein, confers abiotic stress resistance in foxtail millet. BMC Plant Biol.

[15] Huang X, Guo W, Yang L, Zou Z, Zhang X, Addo-Danso SD (2023). Effects of drought stress on non-structural carbohydrates in different organs of *Cunninghamia lanceolata*. Plants (Basel).

[16] Zhao N, Zhao JH, Li SN, Li B, Lv J, Gao X (2024). The response of endogenous ABA and soluble sugars of *Platycladus orientalis* to drought and post-drought rehydration. Biology.

[17] Zhang XZ, Zheng WJ, Cao XY, Cui XY, Zhao SP, Yu TF (2019). Genomic analysis of stress associated proteins in soybean and the role of GmSAP in abiotic stress responses in Arabidopsis and soybean. Front Plant Sci.

[18] Qian Y, Yu H, Lu S, Bai Y, Meng Y, Chen L (2025). Transcriptome analysis reveals the role of plant hormone signal transduction pathways in the drought stress response of *Hemerocallis middendorffii*. Plants (Basel)..

[19] Wei M, Wang B, Li C, Li X, He C, Li Y (2024). Integrated PacBio SMRT and illumina sequencing uncovers transcriptional and physiological responses to drought stress in whole-plant *Nitraria tangutorum*. Front Genet.

[20] Wang Y, Tong L, Liu H, Li B, Zhang R (2025). Integrated metabolome and transcriptome analysis of maize roots response to different degrees of drought stress. BMC Plant Biol.

[21] Wang Z, Yin S, Wei Y, Chen B, Li W (2025). Transcriptome and WGCNA analysis revealed the molecular mechanism of drought resistance in new sugarcane varieties. Front Plant Sci.

[22] Mulozi L, Vennapusa AR, Elavarthi S, Jacobs OE, Kulkarni KP, Natarajan P (2023). Transcriptome profiling, physiological, and biochemical analyses provide new insights towards drought stress response in sugar maple (*Acer saccharum* marshall) saplings. Front Plant Sci.

[23] Ren J, Guo P, Zhao X, Ma X, Ai X, Wang J (2025). Differential photosynthetic responses to drought stress in peanut varieties: insights from transcriptome profiling and JIP-test analysis. BMC Plant Biol.

[24] Guo C, Mo Z, Chen S, Mu K, Yao S, Luo Q (2025). Study on the physiological mechanism and molecular regulatory network of *Blumea balsamifera* in response to drought stress. BMC Plant Biol.

[25] Liu F, Zhao Y, Wang X, Wang B, Xiao F, He K (2024). Transcriptome analysis reveals regulatory mechanisms of different drought-tolerant Gleditsia sinensis seedlings under drought stress. BMC Genom Data.

[26] Du C, Ni X, Yan M, Meng Q, He J (2025). Physiological and transcriptome analysis reveals the mechanism of *Gymnocarpos przewalskii* response to drought stress. BMC Plant Biol.

[27] Li Y, Su Z, Lin Y, Xu Z, Bao H, Wang F (2024). Utilizing transcriptomics and metabolomics to unravel key genes and metabolites of maize seedlings in response to drought stress. BMC Plant Biol.

[28] Wang J, Yao L, Hao J, Li C, Li B, Meng Y (2024). Growth properties and metabolomic analysis provide insight into drought tolerance in barley (*Hordeum vulgare* L.). Int J Mol Sci.

[29] Wang P, Wu Z, Chen G, Yu X (2023). Understanding the response in *Pugionium cornutum* (L.) Gaertn. seedling leaves under drought stress using transcriptome and proteome integrated analysis. PeerJ.

[30] Liu M, Liu Y, Hu W, Yin B, Liang B, Li Z (2024). Transcriptome and metabolome analyses reveal the regulatory role of *MdPYL9* in drought resistance in apple. BMC Plant Biol.

[31] Yu Q, Xiong Y, Su X, Xiong Y, Dong Z, Zhao J (2023). Integrating full-length transcriptome and RNA sequencing of Siberian wildrye (*Elymus sibiricus*) to reveal molecular mechanisms in response to drought stress. Plants (Basel).

[32] Janiak A, Kwasniewski M, Sowa M, Gajek K, Żmuda K, Kościelniak J (2018). No time to waste: transcriptome study reveals that drought tolerance in barley may be attributed to stressed-like expression patterns that exist before the occurrence of stress. Front Plant Sci.

[33] Deng Y, Srivastava R, Howell SH (2013). Endoplasmic reticulum (ER) stress response and its physiological roles in plants. Int J Mol Sci.

[34] Thayale Purayil F, Rajashekar B, Kurup SS, Cheruth AJ, Subramaniam S, Hassan Tawfik N (2020). Transcriptome profiling of *Haloxylon persicum* (bunge ex boiss and buhse) an endangered plant species under PEG-induced drought stress. Genes.

[35] Bashir K, Matsui A, Rasheed S, Seki M (2019). Recent advances in the characterization of plant transcriptomes in response to drought, salinity, heat, and cold stress. F1000Res.

[36] Sharma R, Singh G, Bhattacharya S, Singh A (2018). Comparative transcriptome meta-analysis of arabidopsis thaliana under drought and cold stress. PLoS One.

